# *COLQ*-mutation congenital myasthenic syndrome in late adolescence: Case report and review of the literature^☆^

**DOI:** 10.1016/j.heliyon.2023.e19980

**Published:** 2023-09-12

**Authors:** Yatao Yin, Jing Cao, Yuanteng Fan, Yan Xu

**Affiliations:** a Department of Rehabilitation Medicine, Tongji Hospital, Tongji Medical College, Huazhong University of Science and Technology, Wuhan, China; b Department of Neurology, Zhongnan Hospital, Wuhan University, Wuhan, China

**Keywords:** Congenital myasthenic syndrome, *COLQ*, Adolescence, Case report

## Abstract

Congenital myasthenia syndromes (CMS) are a heterogeneous group of hereditary disorders of the neuromuscular junction. The symptoms include fatigue, muscle weakness, ptosis, mastication or swallowing problem, respiratory distress. We present a 42-year-old male patient who was admitted with complaints of paroxysmal limb weakness for 25 years and got repeated apnea crisis due to using AchE inhibitors. We considered this patient to be *COLQ*-related CMS because of two types characteristics. One is the symptom will deteriorate or non-responsive after giving AchE inhibitors and the other is repeated compound action potentials may appear after one current stimulation. At last we confirmed the diagnosis by genetic testing. It is a rare CMS case caused by homozygous mutation in the *COLQ* gene which occurred at late adolescence. Our case demonstrates that for those serum-negative MG patients, CMS gene mutation screening should be considered, especially if the patient has an symptom onset of childhood and adolescence.

## 1 Introduction

Congenital myasthenia syndromes (CMS) are a heterogeneous group of hereditary disorders of the neuromuscular junction that are caused by mutations in proteins involved in the organization, maintenance, function, or modification of the motor endplate. CMS mostly occurs in neonatal/infant and children, but rarely occurs in adolescence [1]. The hallmark symptom is fatigable muscle weakness, ptosis, mastication or swallowing problem, respiratory distress, scoliosis [2].

Endplate acetylcholinesterase(AchE) deficiency is an autosomal recessive inherited CMS caused by mutations in colq，which results in impaired anchoring of cholinesterase in the synaptic space due to denaturation of *COLQ* protein. The cholinesterase in the synaptic space is relatively lacking and the retention time of acetylcholine in the synaptic space is prolonged, which make cholinergic hyperexcitement eventually lead to endplate myopathy [3]. *COLQ*-related CMS have two types characteristics, one is the symptom will be deteriorated or non-responsive after giving AchE inhibitors, while salbutamol and ephedrine have good effect for some patients. The other one is the post-synaptic membrane continues to be excited, repeated compound action potentials (R-CMAP) may appear after one current stimulation [4]. Herein, we will report a rare case of a male with CMS caused by homozygous mutation in the *COLQ* gene which occurred at late adolescence, who got repeated apnea crisis due to using AchE inhibitors.

## 2 Case report

### 2.1 History of present illness

A 42-year-old male patient was admitted with complaints of paroxysmal limb weakness for 25 years. When the patient was 17 years old, he had weakness in both lower limbs for the first time, he couldn't walk stable, fall down occasionally, and could not stand up after squatting down. Paroxysmal limb weakness occurred intermittently in the past 20 years, which can be induced by cold and fatigue, and can be completely relieved after resting. He was diagnosed of myasthenia gravis (MG) in other hospital. After giving pyridostigmine bromide and a small dose of hormone, the muscle strength was slightly relieved in the first week, Later the weakness symptoms aggravated sharply because of the pneumonia. The patient developed apnea crisis, and was transferred to the Intensive Care Unit for the next 2 months. A tracheotomy was done and he was treated with IVIG, and then he recovered gradually, but rapidly apnea crisis reappeared again.

### 2.2 Previous history and physical examination

The patient had a scoliosis shortly after birth, walked with his head down spontaneously when he was young. His shoulders were tilted when he was 5 years old, and his sports performance was moderate when he was young. His two cousins had a history of paroxysmal limb paralysis and were relieved by Chinese medicine treatment.

Physical examination revealed bilateral ptosis and ophthalmoplegia, scolisis, oblique shoulder and pigeon chest. On strength testing, the deltoids, biceps, triceps, and hip flexors were 3/5 bilaterally; finger/wrist/knee flexors and extensors, foot dorsiflexion, plantar flexion and hand intrinsics were 4/5 bilaterally. The muscle tension was reduced and the reflexes were bilateral symmetric. No obvious atrophy of bilateral muscles was noted. He had dyspnea, \the oxygen saturation was 98% with nasal catheter oxygen inhalation.

### 2.3 The process of diagnosis and treatment

**Fig. 1:**
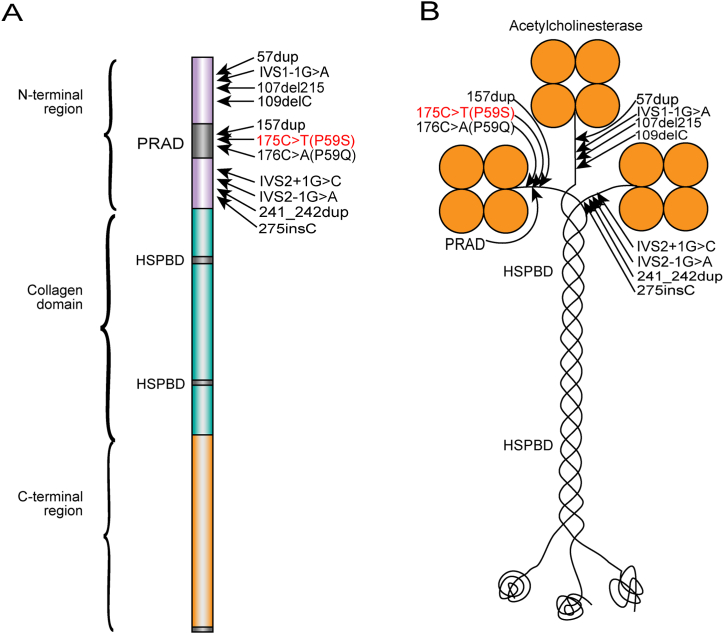
Schematic diagram of a ColQ strand (A) and of the A12 species of acetylcholinesterase (B) with the 11 published pathogenic variants mutations in the N-terminal region and the mutation described in this study (red). PRAD = proline-rich attachment domain. HSPBD = heparan sulfate proteoglycan binding domain.

His neuromuscular junction antibodies LRP4, AchR, MuSK and Titin of serum were negative. Because of his weakness, we increased the pyridostigmine 60mg Q6h to 60mg Q4h, which worsened his weakness. Therefore, we did motor nerve conduction test in electromyogram and found the right median nerve and bilateral tibial nerves showed R-CMAP (74.4% decreasing in the second CMAP compare to the first). So we thought *COLQ*-mutation congenital myasthenic syndrome and slow channel syndrome are most likely diagnosis. Afterward, we stopped the administration of pyridostigmine and gave him salbutamol 4mg bid and fluoxetine 20mg qd for two subtypes of possible CMS. About 2 weeks later, his weakness improved and could walk normally and had normal muscle strength. The whole exome sequence and Sanger sequencing test revealed homozygous pathogenic mutation, C.175C＞T（p.pro59ser, P59S）in *COLQ* gene a month later，as shown in Fig. 1(A、B). So we stopped administration of fluoxetine and just gave him salbutamol 4mg bid. In the last year the patient felt well and not get worse in winter.

## 3 Discussion

*COLQ* mutations cause endplate AchE deficiency is a relatively common type of CMS, which rank only second to the acetylcholine receptor gene *CHRNE* and the receptor-associated synaptic protein *RAPSN* gene mutations. It accounts for about 10–15% of CMS [5]. The endplate species of AchE is a heteromeric asymmetric enzyme composed of one, two, or three homotetramers of globular catalytic subunits (AchET) anchored to the synaptic basal lamina by ColQ, a triple-stranded collagenic tail(Fig. 1B). ColQ was encoded by the gene *COLQ* and contained an N-terminal proline-rich attachment domain(PRAD), a collagenic central domain, and a C-terminal region enriched in charged residues and cysteines(Fig. 1A). Each ColQ strand can bind an AchET tetramer to its PRAD [5] and form a tight coiled-coil assembly in which four alpha helical T-peptides form a cylinder around a PRAD [6]. Mutations in the N-terminal domain prevent the collagen domain from associating with the catalytic subunits [2].

It is the first time reported that the P59S(Fig. 1A and B) of homozygous mutation can lead to *COLQ*-CMS, just compound heterozygous mutations（P59S and C451S）have been reported [7](Table .1). As an important site in PRAD, the mutation replaces proline in PRAD with glutamine, reduces the amount of proline in PRAD, and may also change the spatial conformation of PRAD, significantly reduces the adhesion to cholinesterase, which led to symptoms of muscle weakness. This phenomenon was verified in the same locus homozygous mutations (P59Q) in some studies [2,8].

**Table 1:** Clinical data of the 6 patients with COLQ mutation which occurred at adolescence were found.

Sex	Age of onset(year)	Family History	Motor Weakness	Respiratory crises	Reaction of AchE inhibitors	Gene
M	12	Y	Y	N	ND	ND [9]
F	12	ND	Y	ND	ND	Y440D and I447 M [10]
M	10		Proximal/axial/distal/neck	N	ND	p.C427C (c.1281C > T) Homozygote [7]
F	10	ND	Proximal/Scoliosis	N	ND	Y430S Homozygous [11]
F	12	Y	Proximal	N	ND	R410W Homozygous [12]
M	17	Y	Proximal/axial/distal/neck	Y	worse	P59Q Homozygous

M，male; F，female; Y，yes; ND，no date; N, no.

On the other hand, as a homozygous mutation in this case, it present in late adolescence, this is very rare. In reviewing the previous cases, only 5 cases which occurred at adolescence were found[ [7,[9], [12], [11], [10]]](Table .1). As shown in Table 1, most of these cases are caused by C-terminal mutations. Previous studies shows that severely affected patients with mutations that abolish enzyme activity present in infancy, while less severely affected patients with residual enzyme activity present in childhood and become disabled later in life [2]. Most mutations in the C-terminal domain reduce ColQ expression or prevent the triple helical assembly of the collagen domain [2], but will not affect the connection between ColQ and cholinesterase, and can keep some functions of ColQ, so only have a relatively light impact on patients. However, the P59S homozygous mutation is found in the PRAD site, we speculated that the ColQ protein with P59S mutation still has several functions, and retains the adhesion to cholinesterase, so there is still a certain amount of cholinesterase in the synaptic space. And the acetylcholine function in the synaptic space can be recovered more quickly to normalsitulation, which is the reason why the patient's symptoms quickly recovered after stopping the AchE inhibitor. Although our patient was soon given salbutamol, yet the effect of salbutamol took several months to appear. Therefore, it can be speculated that AchE inhibitors have a significant effect on worsening symptoms in similar patients. This is closely related to another clinical feature of the patient. The patient repeatedly developed muscle weakness symptoms in winter. When the temperature decreased, the symptoms gradually appeared. As we know, low temperature can inhibit the activity of AchE, the effect is similar to using AchE inhibitors and this is consistent with the principle of the ice water experiment [13], which can also explain that the P59S mutation still retains certain cholinesterase function in endplate from the side.

Another article also reported a case with homozygous mutations in P59Q which occurred at 2 years old, but most of the data were incomplete [8]. The same mutations can cause the different onset age and different severity of clinical symptoms. It has also been found in other mutation subtypes and families of *COLQ*. In the patients with Tyr430Ser homozygous mutation, it can be found that the onset age ranged from 1 to 10 years old [11](Table .1). Although the patients in this article did not find any muscle weakness before 17 years old, yet the patient had scoliosis and pigeon chest which occurred earlier than the weakness syndrome. It has been found that some CMS patients may have severe scoliosis[ [11,14]](Table .1)，and microcephaly has also been reported [15]. The skeletal dysplasia will have an important role in the diagnosis of CMS, especially in adult patients who was misdiagnosed of MG, because these changes are not available on MG. This patient has severe scoliosis accompanied by pigeon breast. Scoliosis is found in many cases, but the pigeon breast is rarely reported in previous articles. As a clinical sign with obvious genetic factors, it should be paid more attention.

Misdiagnosis occurred in 94% of the adult patients with CMS. Despite the average age at symptom onset is 1 year, the diagnosis of CMS in the pediatric population was usually delayed to the average age of 4 years [16]. Compared with children's CMS, adult CMS patients have mild weakness, so the diagnostic delay is longer. A retrospective study of the Mayo Clinic's found that the average delay of adult CMS is 30 years [17]. Our patient was also diagnosed 25 years after symptom onset, and due to the inappropriate diagnosis and treatment, repeated crisis were occurred. So it has extremely important clinical significance with the correct diagnosis of CMS in adults with muscular weakness. For those serum-negative MG patients, CMS gene mutation screening should be considered, especially if the patient has an symptom onset of childhood, a positive family history, R-CMAP or the symptoms aggravated or lack of beneficial effects by using pyridostigmine, the skeletal dysplasia such as scoliosis and pigeon breast.

## 4 Conclusion

In this report, we first time reported the P59S of homozygous mutation can lead to *COLQ*-CMS, which is presented in late adolescence. Timely diagnosis, decidedly removal of the AchE inhibitors and reversed myasthenia rapidly. For those serum-negative MG patients, CMS should be considered as an important differential diagnosis.

## Ethics approval and consent to participate

This study has been reviewed and approved by the medical ethics committee, Zhongnan hospital of Wuhan University (approval No.2022033K). Informed consent was obtained from the patient for publication of their clinical data.

## Author contribution statement

All authors listed have significantly contributed to the investigation, development and writing of this article.

## Data availability statement

Data associated with this study has been deposited at Chinese Medical Case Repository; ID：CMCR 2022-01968.

## Declaration of competing interest

The authors declare that they have no known competing financial interests or personal relationships that could have appeared to influence the work reported in this paper.

## References

[1] Finsterer J. (2019). Congenital myasthenic syndromes. Orphanet J. Rare Dis..

[2] Engel A.G., Shen X.M., Selcen D., Sine S.M. (2015). Congenital myasthenic syndromes: pathogenesis, diagnosis, and treatment. Lancet Neurol..

[3] Legay C. (2018). Congenital myasthenic syndromes with acetylcholinesterase deficiency, the pathophysiological mechanisms. Ann. N. Y. Acad. Sci..

[4] Ding Q., Shen D., Dai Y., Hu Y., Guan Y., Liu M. (2018). Mechanism hypotheses for the electrophysiological manifestations of two cases of endplate acetylcholinesterase deficiency related congenital myasthenic syndrome. J. Clin. Neurosci..

[5] Abicht A, Müller JS, Lochmüller H. Congenital Myasthenic Syndromes Overview.. 20301347

[6] Massoulie J., Millard C.B. (2009). Cholinesterases and the basal lamina at vertebrate neuromuscular junctions. Curr. Opin. Pharmacol..

[7] Wargon I., Richard P., Kuntzer T., Sternberg D., Nafissi S., Gaudon K. (2012). Long-term follow-up of patients with congenital myasthenic syndrome caused by COLQ mutations. Neuromuscul. Disord. : NMD.

[8] Mihaylova V., Muller J.S., Vilchez J.J., Salih M.A., Kabiraj M.M., D'Amico A. (2008). Clinical and molecular genetic findings in COLQ-mutant congenital myasthenic syndromes. Brain : J. Neurol..

[9] Wadwekar V., Nair S.S., Tandon V., Kuruvilla A., Nair M. (2020). Congenital myasthenic syndrome: ten years clinical experience from a quaternary care south-Indian hospital. J. Clin. Neurosci..

[10] Engel A.G. (2012). Current status of the congenital myasthenic syndromes. Neuromuscul. Disord.: NMD.

[11] Natera-de Benito D., Topf A., Vilchez J.J., Gonzalez-Quereda L., Dominguez-Carral J., Diaz-Manera J. (2017). Molecular characterization of congenital myasthenic syndromes in Spain. Neuromuscul. Disord.: NMD.

[12] Selvam P., Arunachal G., Danda S., Chapla A., Sivadasan A., Alexander M. (2018). Congenital myasthenic syndrome: spectrum of mutations in an Indian cohort. J. Clin. Neuromuscul. Dis..

[13] Almeida D.F., Radaeli Rde F., Melo A.C. (2008). Ice pack test in the diagnosis of myasthenia gravis. Arquivos de Neuro-Psiquiatria.

[14] Duran G.S., Uzunhan T.A., Ekici B., Citak A., Aydinli N., Caliskan M. (2013). Severe scoliosis in a patient with COLQ mutation and congenital myasthenic syndrome: a clue for diagnosis. Acta Neurol. Belg..

[15] Al-Muhaizea M.A., Al-Mobarak S.B. (2017). COLQ-mutant congenital myasthenic syndrome with microcephaly: a unique case with literature review. Transl. Neurosci..

[16] Kinali M., Beeson D., Pitt M.C., Jungbluth H., Simonds A.K., Aloysius A. (2008). Congenital myasthenic syndromes in childhood: diagnostic and management challenges. J. Neuroimmunol..

[17] Kao J.C., Milone M., Selcen D., Shen X.M., Engel A.G., Liewluck T. (2018). Congenital myasthenic syndromes in adult neurology clinic: a long road to diagnosis and therapy. Neurology.

