# Supplementary material for: COLQ-mutation congenital myasthenic syndrome in late adolescence: Case report and review of the literature
Source: Heliyon. 2023 Sep 12;9(9):e19980. doi: 10.1016/j.heliyon.2023.e19980 (PMC10559664; doi:10.1016/j.heliyon.2023.e19980)
Supplement: Multimedia component 1 [file mmc1.docx]

Ethics approval and consent to participate

This study has been reviewed and approved by the medical ethics committee, Zhongnan hospital of Wuhan University (approval No.2022033K). Informed consent was obtained from the patient for publication of their clinical data.
